# A framework for precision “dosing” of mental healthcare services: algorithm development and clinical pilot

**DOI:** 10.1186/s13033-023-00581-y

**Published:** 2023-07-05

**Authors:** Jonathan Knights, Victoria Bangieva, Michela Passoni, Macayla L. Donegan, Jacob Shen, Audrey Klein, Justin Baker, Holly DuBois

**Affiliations:** Mindstrong, Inc., 101 Jefferson Drive, Suite 228, Menlo Park, CA 94025 USA

**Keywords:** Mental healthcare, Systems dynamics, Indirect response models, Digital healthcare, Telehealth, Learning healthcare system, Measurement-based care, Precision medicine

## Abstract

**Background:**

One in five adults in the US experience mental illness and over half of these adults do not receive treatment. In addition to the access gap, few innovations have been reported for ensuring the right level of mental healthcare service is available at the right time for individual patients.

**Methods:**

Historical observational clinical data was leveraged from a virtual healthcare system. We conceptualize mental healthcare services themselves as therapeutic interventions and develop a prototype computational framework to estimate their potential longitudinal impacts on depressive symptom severity, which is then used to assess new treatment schedules and delivered to clinicians via a dashboard. We operationally define this process as “*session dosing*”: 497 patients who started treatment with severe symptoms of depression between November 2020 and October 2021 were used for modeling. Subsequently, 22 mental health providers participated in a 5-week clinical quality improvement (QI) pilot, where they utilized the prototype dashboard in treatment planning with 126 patients.

**Results:**

The developed framework was able to resolve patient symptom fluctuations from their treatment schedules: 77% of the modeling dataset fit criteria for using the individual fits for subsequent clinical planning where five anecdotal profile types were identified that presented different clinical opportunities. Based on initial quality thresholds for model fits, 88% of those individuals were identified as adequate for session optimization planning using the developed dashboard, while 12% supported more thorough treatment planning (e.g. different treatment modalities). In the clinical pilot, 90% of clinicians reported using the dashboard a few times or more per member. Although most clinicians (67.5%) either rarely or never used the dashboard to change session types, numerous other discussions were enabled, and opportunities for automating session recommendations were identified.

**Conclusions:**

It is possible to model and identify the extent to which mental healthcare services can resolve depressive symptom severity fluctuations. Implementation of one such prototype framework in a real-world clinic represents an advancement in mental healthcare treatment planning; however, investigations to assess which clinical endpoints are impacted by this technology, and the best way to incorporate such frameworks into clinical workflows, are needed and are actively being pursued.

**Supplementary Information:**

The online version contains supplementary material available at 10.1186/s13033-023-00581-y.

## Background

It is estimated that 1 in 5 adults in the United States (US) experience mental illness [1]. In 2020 alone, the US government spent roughly $280B on services for mental health and substance use disorders [2]. Despite this figure, the percentage of adults with unmet needs has been increasing annually since 2011 and over half of US adults with mental illness do not receive treatment [3]. At the current pace, provider shortage is projected to increase reaching a shortage of 30,000 psychiatrists within the next few years [4]. In addition to the access gap, treatment planning and evaluation within the psychotherapy landscape is still largely conducted in the absence of measurement and data, leading to significant inefficiencies and treatment quality concerns [5, 6]. This evidence supports the need for novel mental health treatment models and technologies focused on streamlining the planning and management of care to help address the persistent mental healthcare crisis: For example, in a recent article exploring opportunities for optimizing personalized management of depression, Reynolds [7] highlights “… How to leverage mental health expertise broadly in the service of personalized prevention and treatment, [therefore] becomes the central question.”

From a technology standpoint, there has been an uptick in the application of machine learning and artificial intelligence (ML/AI) to improve access to—and the effectiveness of—mental healthcare via chatbots and predictions for the most appropriate medications or treatment courses [8–15]. While some hypothesize that chatbots may reduce the need for trained mental healthcare professionals from the process of delivering care, a 2020 survey of global psychiatrists [16] found that only 7 of 792 (0.9%) believe AI agents will be able to provide empathetic care. Current technological innovations exist predominantly in the medication and care delivery spaces, with little to no focus on how individuals interact with mental healthcare services as a type of therapeutic intervention in and of itself—presumably due to a lack of historical data enabling such investigation. Therefore, at least in the near term, impactful technological developments in mental healthcare will likely require interactions with trained professionals (humans in the loop), and there is a need for innovation in the domain of optimizing resource allocation in a more efficient and precise fashion.

Some current innovative clinical frameworks for triaging services as part of a patient’s treatment plan involve a stepped care model, which advocates for the care of individuals by optimally utilizing scarce resources to their greatest effect at the population level [17–19]. The “stepped” interventions are typically determined by an individuals’ presenting problems, preferences, and ideally clinical outcomes [19]. However, treatment decision protocols can be subjective and lack a holistic assessment of all possible data, rendering them largely unsuitable for objective personalization or real-time adjustments. Further, it has been noted that implementation of stepped care methods can vary greatly across institutions [20]. While clinical judgment is a valuable component to treatment decisions, it is not always accurate - providers tend to overestimate their success with patients relative to other providers, and they are not always effective at identifying when patients are getting worse due to a positive self-assessment bias [21]. Given the increased adoption of technology in mental healthcare (which has been amplified by the COVID-19 pandemic [22–24]) and increased attention on clinically relevant passive and active measurement [25–28]—enabled by increased adoption of mobile technologies—data is emerging to enable objective, near real-time, personalization of treatment needs, as well as service and resource optimization.

To begin addressing the above gaps, in this work, we present a prototype computational framework for optimizing mental healthcare services at the individual level, as well as preliminary findings from an exploratory pilot deploying this framework in the clinic. Historical real-world (observational) clinical data was leveraged from a virtual mental healthcare platform, which focuses on measurement-based care and captures routine patient-reported symptom severity reports in between individual mental healthcare sessions. By conceptualizing psychotherapy and supporting service appointments as ‘therapeutic interventions’ and modifying quantitative approaches rooted in pharmacodynamics [29], we estimate temporal impacts of individual services on depressive symptom severity at the population and individual patient levels. The proposed framework is subsequently able to generate hypothetical symptom severity trajectories based on new potential treatment schedules. We operationally define this process as “*session dosing*” and describe some of the technical aspects of the quantitative framework, as well as interpretive observations, strengths, and limitations, through real patient vignettes. Finally, we present preliminary learnings and clinician feedback from a pilot in which we deployed this framework to clinicians as an exploratory clinical quality improvement (QI) initiative. To our knowledge, this work is the first proof-of-concept for the application of quantitative science on mental health symptom data to identify impacts of individual services at the patient level, which can then be applied to optimize mental health service utilization.

## Methods

### Observational cohort and analysis data

Retrospective observational clinical data, generated from real-world mental healthcare treatment at Mindstrong was queried October 1, 2022, for individuals who screened into treatment with a severe symptom severity report based on the DSM-5 Level 1 cross-cutting symptom self-report questionnaire [30] (DSM-L1). Although not a standard research symptom severity measure, patients within the Mindstrong healthcare system predominantly represent individuals suffering from serious mental health conditions, most of which are marked by symptoms that reach across problem domains. For this healthcare system, the choice of the DSM-L1 was intentionally rooted in this concept - an ability to monitor patients via their chronic conditions, rather than a singular diagnostic tool. As part of routine clinical care, patients were administered the DSM-L1 questionnaire at regular intervals (every 60 days prior to September 2021; every 30 days after September 2021).

Depressive symptoms in the DSM-L1 are captured by two questions that utilize a 5-point Likert scale to evaluate how much, or how often, an individual has been bothered by that symptom during the last 2 weeks (0 = not at all/none, 1 = rare, less than a day or two/slight; 2 = several days/mild, 3 = more than half the days/moderate; 4 = nearly every day/severe): Item 1 measures anhedonia, which prompts “how often have you had little interest or pleasure in doing things?”, while item 2 measures depressed mood, which prompts “how often have you been feeling down, depressed, or hopeless?” An average of the two depression items was taken as the single measure by which to represent depressive symptom severity at a given time point. This value was subsequently normalized by the maximum possible value—4 in this case—to generate a value in the range [0,1]. It is acknowledged that representing—and modeling—depressive symptom severity from these two items alone does not capture the full spectrum of depressive symptoms; however, these items are integral to the measurement-based care model of the presented healthcare system and form the foundation by which clinicians who operate in this system track symptom severity over time. As will be discussed later, the presented framework is conceptually agnostic to the underlying measures driving the symptom severity score, and so while the results are intended only to pertain to the healthcare system of interest, the concepts and novel opportunities for clinical decision making are innovations that could benefit the mental healthcare field at large.

The minimum criteria for inclusion in the analysis data set was to have at least two DSM-L1 reports, with at least one treatment session (of any kind) in between those reports. The available treatment session types were: (i) coaching sessions with an unlicensed professional, which focus on social determinants of health issues and assistance with planning, organization, and resource connection; (ii) therapy sessions with a licensed therapist or counselor; (iii) psychiatry sessions for treatment and medication management. There were no additional demographic or clinical inclusion/exclusion criteria assigned for the analysis. The specific data elements leveraged were the timing and type of treatment sessions, and the timing of depressive symptom reports within the sample window.

A total of 497 patients were included in the modeling analysis. Table 1 provides a breakdown of the clinical and demographic factors of the patients present in this analysis, while Table 2 presents a summary of the amount, and duration, of data present for the sample population. All individuals were initially referred as eligible for care by their insurance providers and incurred no additional out-of-pocket costs for receiving services.

**Table 1:** Analysis sample population demographics

	N (% Total)
Age (years)	
< 30	15 (3.0)
30–39	37 (7.4)
40–49	95 (19.1)
50–59	155 (31.2)
60–69	153 (30.8)
70+	42 (8.5)
Gender	
Female	359 (72.2)
Male	135 (27.2)
Unknown	3 (0.6)
Race/Ethnicity	
White	251 (50.5)
Black or African American	38 (7.6)
Hispanic or Latino	30 (6.0)
Other	26 (5.2)
Unknown	152 (30.6)
Living area	
Rural	282 (56.7)
Urban	215 (43.3)
Primary diagnosis	
Bipolar disorder	98 (19.7)
Major depressive disorder	241 (48.5)
Personality disorder	33 (6.6)
Schizophrenia or related	24 (4.8)
Other	101 (20.3)

**Table 2:** Summary of historical treatment sessions and correlations between service types

Observation type	Coaching	Therapy	Psychiatry	DSM-L1 reports
Count	851	8043	1746	3107
Average per patient (mean)	1.7	16.2	3.5	6.2
Standard Deviation	4.8	15.3	5.2	4.5
[Min., Max.]	[0, 44]	[0, 84]	[0, 33]	[2, 22]
Quartile cutoffs [25%, 50%, 75%]	[0, 0, 0]	[4, 11, 25]	[0, 1, 5]	[3, 5, 8]
Correlation matrix for observation types				
Coaching	1			
Therapy	0.12	1		
Psychiatry	0.19	0.41	1	
DSM-L1 Reports	0.12	0.72	0.45	1

### Modeling framework

The data leveraged comprises real-world clinical schedules not designed a priori. Successive treatment sessions may occur at any time interval relative to each other, or relative to each symptom severity report in the sample window. For this type of analysis, we employed a general systems dynamics modeling framework. Specifically, we draw inspiration from the field of pharmacometrics and adapt the application of indirect response (IDR) models [29], conceptualizing a clinical session (treatment) as a type of therapeutic intervention that carries some theoretical ‘therapeutic mass’, which can positively impact symptom severity levels. Traditionally, IDR models are used to represent interventions on biological processes that have natural turnover, whose production or elimination rate is the target of the intervention. In these scenarios, IDR models resolve the time delay between intervention and subsequent biological impact: Some examples (not exhaustive) from the literature can be found in the fields of oncology [31–33], hematology [34–36], and rheumatology [37–39].

The IDR models contrast to direct effect models, which assume an immediate impact on the dependent variable. Previous internal exploratory modeling (results not shown) did not support this approach as a better fit for the available data; however, as will be discussed more in subsequent sections of this report, it is emphasized that the proposed framework is a prototype that fits the requirements of the problem, and opens the door for further research, rather than a formal declaration of model propriety. Indeed, it will be warranted to continually evaluate different modeling hypotheses and use cases, as well as allowing for the possibility that subgroups may exist in the population which align more to the assumptions of one model over another.

**Fig. 1:**
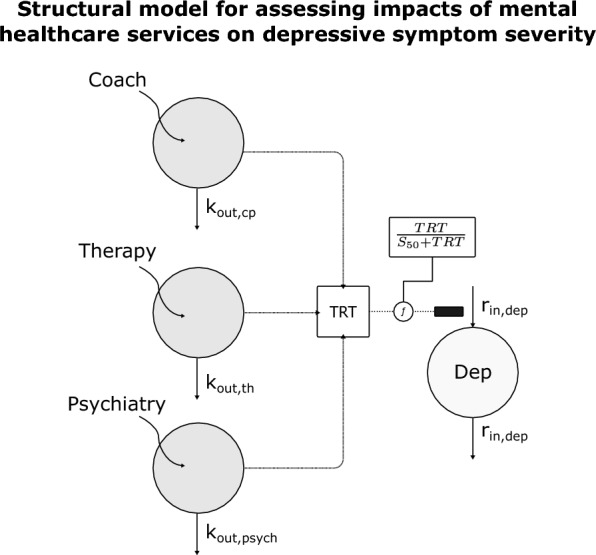
Indirect response model structure employed to capture population dynamics of responses to different types of mental healthcare services. The structural model is set up to take session events as unit inputs into a latent service compartment. The choice of using a unit input as 'mass' into the latent space is arbitrary and can be challenged with the data over the course of time to gauge improvements in the fit of the data. Each service compartment has a first-order elimination rate constant (${k}_{out, *}$), which is a convenient starting point for modeling as it ensures values greater than zero. Each service compartment is combined into a combined latent treatment 'mass' (TRT), which is used to drive inhibition of depressive symptoms. The function driving the inhibition of symptom generation is a type of Hill function with a capacity/sensitivity term (${S}_{50}$) driving the duration of successful inhibition of symptoms. The proposed model also makes the simplifying assumption that the maximum effect term in the usual Hill function be fixed to 1 and is therefore omitted in the expression. The interested technical reader may refer to Additional file 1: S1 for additional details of the model. For this work, only members who enter treatment with severe depression were included. Depression symptom dynamics (“Dep” compartment) are modeled as having zero-order production and first-order elimination. The values of this compartment are patient-reported symptom severity scores. A further simplifying assumption is made so that ${r}_{out,Dep}={r}_{in,Dep}$, which allows the change in symptoms to be capped at the severe ($Dep=1$) level

For this work, depressive symptoms are represented to be at a certain equilibrium at onset, with symptoms being generated and resolved naturally (although at elevated levels)—treatment (session utilization) impact is assumed to decrease symptom severity and is parameterized to decrease the production of symptoms. An analogous effect on symptom severity was explored, parameterizing the treatment effect as leading to an increase in the elimination rate of symptoms (results not shown); however, this approach required the addition of an unbounded ‘stimulation’ (${S}_{max}$) parameter to the Hill function in the model and led to numerical difficulties in the estimation process. It was for these reasons that the simplified, bounded, nature of parameterizing the treatment effect as reducing (potentially completely turning off) the symptom generating process was adopted. Figure 1 shows the base structural model—further technical details of the model, including the statistical error model assumptions, can be found in the Additional file 1: S1. Model parameter estimation was completed leveraging the stochastic approximation expectation–maximization (SAEM) [40] algorithm utilizing the nonlinear mixed-effects modeling software, NONMEM (see below)—the model code is available as Additional file 2: S2).

It is possible that analytical approaches outside of general systems dynamics frameworks may be capable of modeling this type of data; however, the current approach was selected given our group’s expertise in this domain, its ability to accommodate sparse, asynchronous, and irregularly timed observations, as well as its appropriateness for capturing individual and population expectations with physiologically motivated systems. It must be emphasized that this framework is not intended to be, nor is equipped to be, mechanistic or deterministic—it was developed specifically to accommodate real-world healthcare systems with sparse and irregular data availability. Rather than attempting to generate consensus on the most appropriate modeling approach, its primary objective is to provide one possible quantitative framework by which discrepancies and likenesses of individual data fits can be objectively evaluated and used to triage clinical decision making.

### Determination of support level for individual model fits

It is not the expectation that the assumptions of the proposed model should support every individual’s data in the same fashion: The sparsity of the data, combined with the implicit assumption that session schedules alone will resolve symptom severity trajectories for the whole population, do not support such an expectation. In order to identify the potential treatment planning options the framework may be capable of supporting for each individual, it is necessary to establish an objective approach for determining how well the model fits each individual’s data at each iteration. In this prototype, assessment of the level of confidence (support) for the model fit at an individual level was computed using the individual weighted residuals (IWRES: defined below): Smaller IWRES descriptives at the individual level indicate better model fits, meaning that those individual symptom dynamics profiles were better explained by historical treatment schedules (and the assumptions of the model), while larger IWRES descriptives at the individual level indicate that the symptom dynamics profiles may not be well- resolved by the assumptions of the model and may need to be triaged separately.

IWRES weights individual observed residuals ($RES=observed-predicted$) by the defined population error model. In the case of the current model, a simple additive residual error model was employed, which simply weights $RES$ values by the standard deviation of the observed residual variability, turning it into a type of z-score. Details for the interested technical reader can be found in the Additional file 3: S3.

All TIME = 0 observations are removed from support determination as these residuals are always zero given the way the symptom severity component of the model is initialized. Therefore, individuals who had the minimum number of data points necessary to be in the model (two symptom reports and at least one session in between) are not able to be classified for support: By definition, these individuals would not be supported in the sense that there is not enough data to have confidence in the quality of the model fit for treatment planning. However, they do provide information for the model at the population level, which is why they remain in the sample modeling population. Table 3 highlights the initial logical thresholds for determining the level of support the model fit has for the individual: These values were chosen from practical considerations and empirical inspection of initial model fits and will require further optimization in the future as specific outcome targets become of interest (e.g. outcome specific thresholds).

**Table 3:** Logical rules for determining support level for individual model fits

Supported	$ABS\left(\overline{IWRES}\right)\le 1$	${\sigma }_{IWRES}\le 1.5$
Semi-supported	$1<ABS\left(\overline{IWRES }\right)\le 1.5$	$1.5<{\sigma }_{IWRES}\le 2$
Unsupported	$ABS\left(\overline{IWRES }\right)>1.5$	${\sigma }_{IWRES}>2$

ABS: absolute value; $\overline{IWRES }$: observed average IWRES for individual; ${\sigma }_{IWRES}$: observed standard deviation of IWRES for individual

The approach of conducting model evaluation at the individual level based on estimated fits from all available data diverges from more traditional model evaluation strategies leveraging performance metrics such as sensitivity, specify, predictive power, or cross-validation. Diverging from these traditional performance metrics is necessary in this approach for two primary reasons—the first being that models should be evaluated against their intended function. For instance, one would not evaluate predictive power for a model that was not generating predictions. In this case, the function of the model is to assess the strength of evidence supporting a link between treatment schedules and clinical symptom trajectories, which requires inclusion of all available data as well as evaluation of the model fits at the individual level. The second reason for diverging from traditional model evaluation metrics with this framework is that the application of the model to inform clinical practice (the intended function) creates a non-static data generation process: The application of these model fits to impact treatment planning may change a number of behaviors that could fundamentally alter the relationship between treatment schedules and symptom severity trajectories. For instance, after identifying a patient whose treatment schedule does not appear to impact their symptom trajectory (“unsupported” scenario in Table 3), the clinical team may decide to adopt a new treatment modality, which may improve the link between treatment schedules and symptom reports. This phenomenon means that ‘performance’ of the model in the traditional context is not expected to remain constant and reporting momentary snapshot performance is misleading. It is worth noting, methodologically, that performance metrics tracking changes in model fit over time, or improving treatment efficiency measures, will be critical evaluation strategies—closer to traditional model performance—when the framework is deployed in clinics for longer durations.

### Clinical beta test pilot

A qualitative, exploratory, clinical quality improvement pilot was executed over 5 weeks to investigate early potential implementation pitfalls for the modeling framework and pressure test assumptions of clinical utility in patient care and care operations.

To be eligible for participation, a clinician must have had active patients on their caseload who entered treatment (intake assessment) with a severity score for either anxiety or depression of “severe” via the DSM-L1. A total of 23 clinicians were initially selected from the mental health professionals employed at Mindstrong. One clinician transferred all their patients to a new provider halfway through the pilot, but was still able to complete the evaluation questionnaire. Another clinician took a leave of absence and was unable to engage in the pilot, resulting in 22 provider participants, including 17 therapists, 3 coaches, and 2 psychiatric prescribers. A total of 126 patients were included in the pilot. Note that this set of patients constituted active patients and is a subset of the analysis data set used for the modeling work, which included inactive and historical patient data as well.

All clinicians attended an initial instructional meeting that included conceptual case studies for exploration on how to apply the modeling framework in clinical practice. Following the initial training, clinicians were given a resource guide and had access to the full technical and clinical teams for support. Over 5 weeks, clinicians were asked to utilize a secure “Session Dosing” dashboard similar to the concept mock-ups in Fig. 5 prior to each session with an eligible patient. Clinicians were asked to record changes in session frequency, as well as their rationale for the decision. Finally, they were also asked to discuss suggested changes with their patients as part of their treatment planning.

Clinicians provided feedback in two 30-min internal consultation meetings (weeks 1 and 3) and completed a 38-item questionnaire (Google form) at the end of the pilot (week 5). Consultation meetings were informal and were an opportunity to provide feedback and ask questions. The 38-item evaluation questionnaire was developed and reviewed by a cross-functional team to evaluate the pilot and is available in Additional file 4: S4.

### Software and hardware

All modeling was conducted using the *NONMEM* software [41] running on an Amazon Web Services (AWS) EC2 instances with 4 compute cores, 16 GB RAM, and 50 GB root volumes. All data processing and plotting were conducted with MacBook Pro computers running 32 GB DDR4 RAM on 2.3 GHz 8-Core Intel Core i9 processors and 1 TB solid state hard-drives, utilizing the Python programming language [42].

The Prototype dashboard was implemented using the “shiny” package [43] in R [44] to create a web application hosted on Amazon AWS. The deployed dashboard was protected with VPN to ensure only participating clinicians could access the web application.

Survey response process and descriptive statistic summaries were conducted with Google Sheets on a MacBook Pro with 16 GB GB DDR4 RAM on 2.3 GHz 8-Core Intel Core i9 processors and a 500 GB solid state hard drive.

## Results

### Observational cohort

Table 1 displays the breakdown of sample demographics for the observational cohort. There was a total of 497 individuals included in the modeling analysis. The sample population was predominantly White (50.5%), Female (72.2%), and mostly aged 50 years and older (70.5%). Roughly 31% of individuals did not have registered ethnicities. A majority of individuals in the sample population were of rural living status (56.7%), which speaks to one of the benefits of virtual mental healthcare. Of the primary diagnoses captured in the sample population, Major Depression was the most predominant (48.5%), which is largely reflective of the analysis condition of including individuals who entered services with severe depressive symptom levels: bipolar disorder was the second most predominate condition (19.7%), while personality disorder and schizophrenia made up 6.6% and 4.8% of the sample population respectively. Data wise, coaching sessions were the least prominent, but these sessions were also the most recent addition to the clinical care offerings. There was a moderately strong positive correlation between therapy and psychiatry utilization in the sample data ($r=0.41$).

### Modeling

**Fig. 2:**
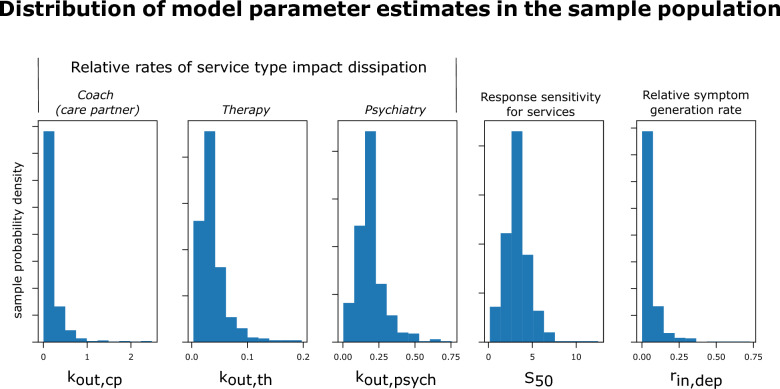
Final model parameter distribution histograms. In the model, sessions represent a ‘therapeutic dose’ (input) and the ${k}_{out,*}$ terms represent how fast that input dissipates in an individual, which would be analogous to how fast something is eliminated from the body—the higher these values, the faster the input dissipates. There is increasing variability, and increasing typical (average, or central tendency in the population) values observed coinciding with the clinical triaging hierarchy of coaching, therapy, and psychiatry. The “response sensitivity for services” (${S}_{50}$) term indicates how much of the therapeutic input is necessary to sustain a response (see Materials & Methods) and demonstrated a range of sensitivities to treatment across an order of magnitude. The “relative symptom generation rate” represents how fast symptoms are observed to return to baseline (severe levels in this case). Note again (from Fig. 1) the simplifying assumption that was made such that ${r}_{out,dep}= {r}_{in,dep}$

Figure 2 shows the distribution of the population estimates of the model parameters in the sample population. It is important to note that the parameters do not have interpretable values—or units—from a clinical perspective as the model space is latent in nature and does not represent physical measurements; however, comparative interpretations of the parameters are appropriate. Having higher- or lower-values for a set of parameters can be interpreted relative to either the average population value for those parameters, or to compare a set of individuals for treatment planning implications.

In this model, sessions represent a ‘therapeutic dose’ (input) and the ${k}_{out,*}$ terms represent how fast that input dissipates in an individual, which would be analogous to how fast a medication was eliminated from the body—the higher these values, the faster the input dissipates. There was increasing variability, and increasing typical values (average, or central tendency, in the population) observed coinciding with the clinical triaging hierarchy of coaching, therapy, and psychiatry. The “response sensitivity for services” (${S}_{50}$) term indicates how much of the therapeutic input is necessary to sustain a response and demonstrated a range of sensitivities to treatment across an order of magnitude for the observed sample window, which is logically consistent with historically observed data of a wide range of response levels to mental healthcare treatment, whether pharmacological, psychosocially focused, or both. The “relative symptom generation rate” represents how fast symptoms are observed to return to baseline (severe levels in this case). While there is some variability in this parameter, the distribution is heavily focused around the population tendency, which may be a result of the homogeneity of the sample from a starting symptom severity level, e.g. all individuals in the analysis sample screened into treatment with severe depressive symptom levels according to the DSM-L1.

The final model fit indicated bias in capturing the lowest symptom levels, e.g. symptom severity reports of ‘none’ (severity = 0), where it systematically predicts higher values for lower observations. This is not a barrier for application as the proposed utility in clinical decision making involves semi-quantitative assessment of symptom changes in the near-term, intended to be reviewed and updated regularly. Rather, this is an important point to note and account for during application and to progress future model development and refinement. More details around this topic are provided in the discussion section. Full model diagnostic plots for sample population are available as Additional file 3: S3 for the interested technical reader.

### Observed example profiles

**Fig. 3:**
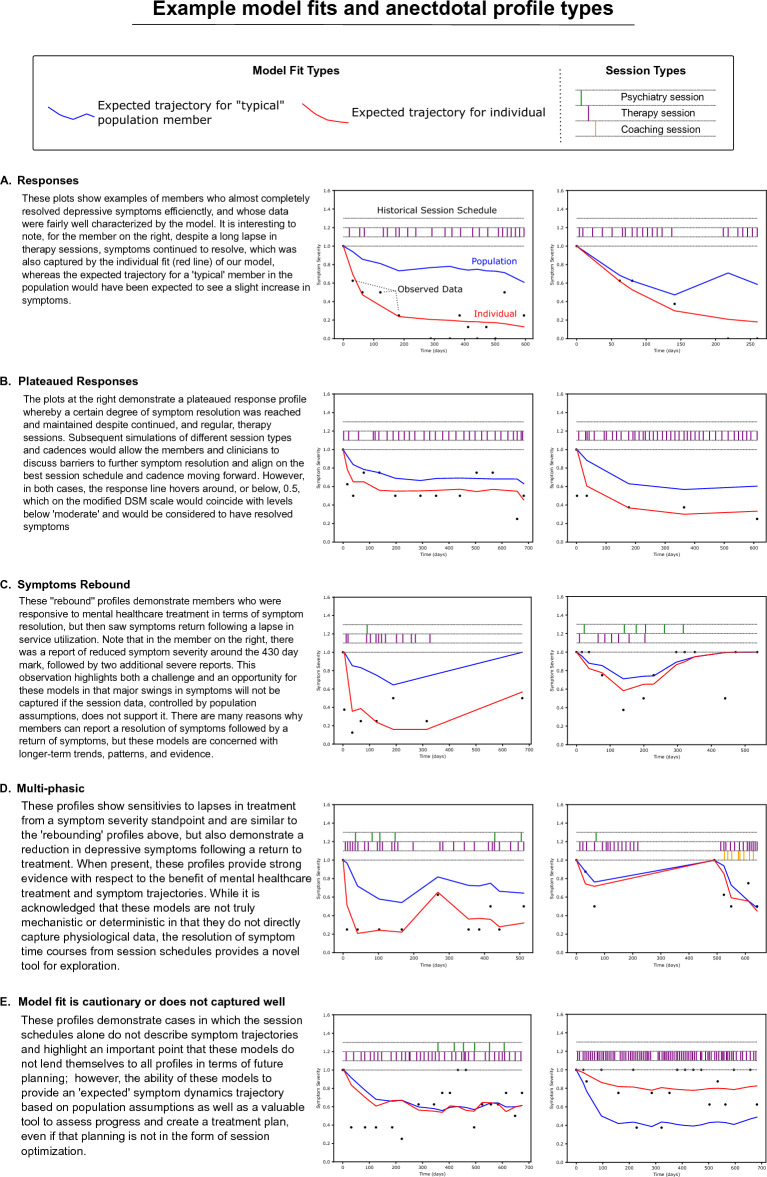
In the figures above, the figure insets represent member (patient) symptom severity reports for depression via the DSM-5 Level 1 cross-cutting symptom survey, requested at regularly scheduled intervals as part of routine care. In this analysis population, all members started treatment with severe reported symptom severity. The black dots represent the reported severity, scaled from 0 to 4 (none-severe) to 0–1. The blue and red lines are model generated predictions for the 'typical' expected population value in the hierarchical model given that members session history (frequency and type), while the red line indicates the member specific predictions. The tracts and line segments above the observed/predicted data show the session cadence for each individual: the yellow line segments at y = 1.0 represent coaching (care partner) sessions, the purple line segments at y = 1.1 represent therapy sessions, and the green line segments starting at y = 1.2 represent psychiatry sessions. The x-axis (time) is presented as days. The different profiles are described in the text inserts in **A**–**E**

Figure 3 shows individual fits of the data by the presented model which have been organized, anecdotally into different types of profiles for the purposes of interpretation and discussion. There are five types of fits highlighted, ranging from a ‘response’ profile to ‘data not well captured’. The profiles highlight the hierarchical nature of the model fits; there is a ‘population’ fit, which is the expected profile for a ‘typical’ individual in the population (meaning the profile expected to be generated for an individual whose parameter values were equal to the population average given the same treatment schedule), as well as an ‘individual’ fit, which results from the optimized set of parameter values for that individual and their historical treatment schedule. An important result of this manuscript is the description of how to interpret the model fits conceptually and how that may relate to treatment planning: When the model fits the data very well, this indicates that the historically reported symptom severity levels can be well characterized by the observed session schedule at the time the model was run. Conversely when the model does not fit the data well, this indicates that the historical symptom severity reports do not appear to be directly linked to the session cadence at that time. In either case, future model runs may demonstrate different levels of confidence, or patterns, relative to what is observed initially. While this result may seem to challenge the utility of the proposed framework, the model provides a tool to assess how tightly mental health symptom severity is associated with healthcare service utilization, in order to make a more informed decision on how to proceed with future care coordination and planning—if there is no historical evidence supporting utility from services received, knowing this early is informative and provides an opportunity to change course. Therefore, it is important to develop and evolve methods of appropriately triaging types of model fits (see Table 3). A summary of the presented model fit types from Fig. 3 is provided below.

*“*Response” profiles (Fig. 3A) demonstrate a high degree of symptom resolution that coincides temporally with historical treatment schedules and whose data are well captured by the model. For these individuals, planning treatment schedules around a maintenance mode could be one possible next step and utilization of the model. “Saturated Response” profiles (Fig. 3B) demonstrate a plateau in symptom resolution—to varying degrees—and in the case of the profiles shown, a fairly regular cadence of treatment touch points. In terms of downstream clinical utilization of the framework for these individuals, an assessment of additional resources or a deeper dive into what would be needed to further resolve symptoms could be had, or it may be determined that the ‘plateaued’ response is adequate from a clinical perspective and a maintenance approach would be warranted. “Rebounding symptom” profiles and “multi-phasic*”* profiles (Fig. 3C, D) are similar in that a lapse in treatment demonstrates a corresponding increase in symptoms, while what distinguishes them is simply a matter of when the lapse took place relative to their historical treatment schedule. In the case of the multi-phasic individuals, an observed period of re-engagement with services resulted in subsequent decrease in symptoms, while the ‘rebound’ profiles did not re-engage with services during the analysis window. It is noteworthy to discuss the left example of Fig. 3D, where the ‘rebound’ phase appears to be uniquely characterized by a single observation between days 200–300, emphasizing that these profiles should not be interpreted as fixed or static types in the population, rather useful vignettes for discussing observed patterns. If that observation was indeed an anomaly, as more data is collected, the less impactful it would be to the overall fit; however, it is also true that the observed spike from low- to moderate-symptom severity coincides with the longest gap in treatment observed for that individual. “Cautionary”, and “not captured well” model fits (Fig. 3E) are also present in the data. This is an important result to be embraced not as a limitation, but as another opportunity to make earlier and more informed clinical decisions. For instance, the lack of relationship between treatment schedules and symptom resolution may indicate individuals requiring more—or different types of—support, or who may require different clinical strategies. Identifying these individuals objectively and early, would be a benefit to any clinical team treating patients. It is noted that there may be very reasonable external factors or challenges at the individual level preventing someone from making sustained progress with their depressive symptoms—or perhaps they are not ready to start addressing their depressive symptoms yet with their clinical team. All these situations are feasible, but without a framework for identifying when symptom trajectories are unexpected, teams are not able to respond accordingly.

Table 4 outlines the breakdown of the sample population in terms of the level of confidence for utilizing the fit for treatment optimization based on this initial approach leveraging individual weighted residuals as a guiding metric (see Materials & Methods, as well as Additional file 3: S3 for supporting figure and description of the approach). Of the 497 individuals in the severe depression sample population, 116 (23%) were excluded from summarizing model fit confidence for not having at least two observations points following their baseline observation. Of the remaining 381, 335 (88%) were considered ‘well supported’, 35 (9%) were considered as “Semi-supported”, and 11 (3%) were considered “Unsupported”.

**Table 4:** Distribution of identified strengths of evidence plots (N = 497)

Classification	N (% total)	% Classified
Supported	335 (67%)	88%
Semi-supported	35 (7%)	9%
Unsupported	11 (2%)	3%
Not enough data to classify^a^	116 (23%)	NA

^a^“Not enough data to classify” implies these individuals met the criteria for modeling, but did not meet the criteria for classifying

### Informing treatment planning

**Fig. 4:**
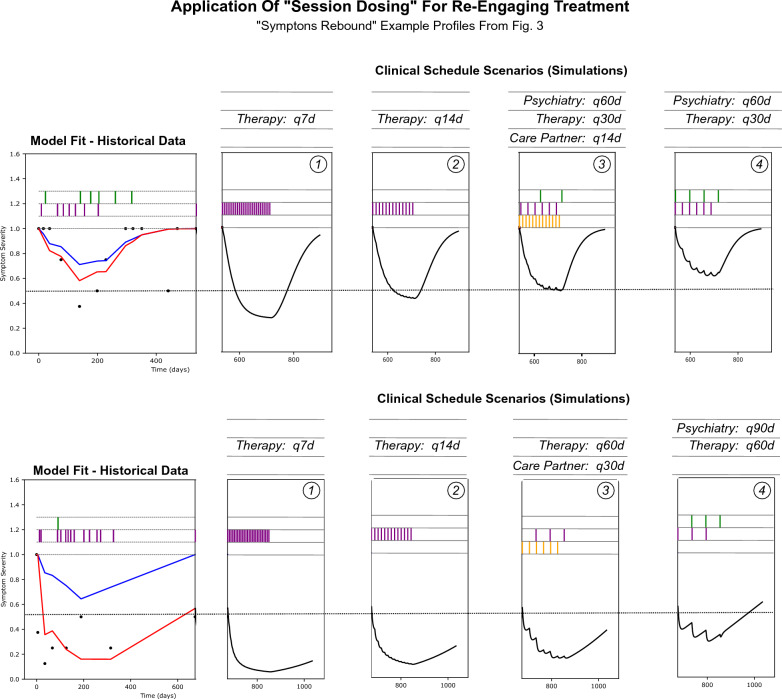
A comparative application of the session 'dosing' model to inform treatment planning for two patients with different sensitivities to lapses in session cadences, based on their historical data. The "q**d" nomenclature indicates schedules at a particular frequency, where 'q7d' translates to every seven days. The observed and model fit plot (far left) is the same as that in Fig. 3. The dashed horizontal line across the image represents a hypothetical 'response' threshold/reference and maps to sub-moderate symptom severity on the DSM L1 survey. All simulations were generated with 180 days of treatment followed by an additional 180 days of no treatment. For the top profile, consistent care appears to be paramount. In all the simulations for this profile, (despite the response magnitude) historical data suggests that this patient will return to a severe level of symptoms within 6 months. Historical data for the bottom profile suggests that this patient would be amenable to less frequent sessions. For these profiles, and all other applications of this framework, as new data is collected the relationships may shift, highlighting that these models should not be applied without review and regular collection of new data

Figure 4 demonstrates how the presented modeling framework may be utilized to inform treatment planning for the two individuals with ‘rebounding symptoms’ in Fig. 3. In these instances, developing a re-engagement schedule informed by historical data appears feasible. In the figure, the dotted horizontal line represents a target that may be set (or moved). The historical data for these two profiles suggests different levels of treatment frequency would be warranted. For instance, therapy every other week may be deemed the most appropriate starting point for the top profile, while monthly coaching sessions along with therapy every other month may be a better starting point for the bottom profile. These individuals started at identical places in terms of depression severity, and subsequently responded differently to their observed historical treatment schedules, which enables the generation of an informed—personalized—treatment schedule for reengagement.

### Clinician feedback and observations from an exploratory clinical quality improvement pilot

**Fig. 5:**
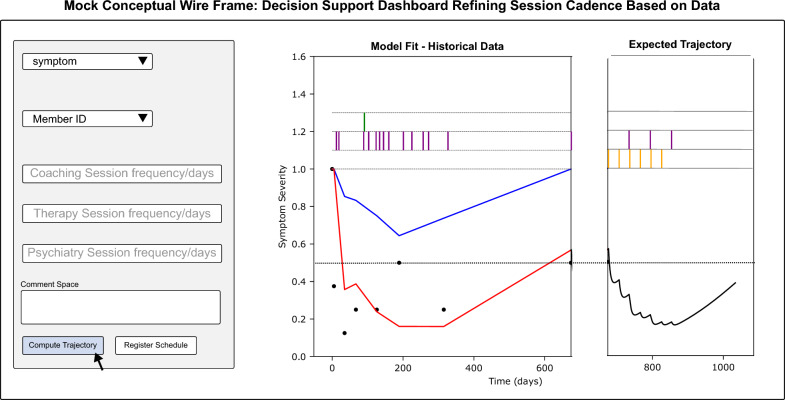
Conceptual mock wireframes for a dashboard intended to facilitate simulation of personalized treatment schedules by clinicians for individual patients

**Fig. 6:**
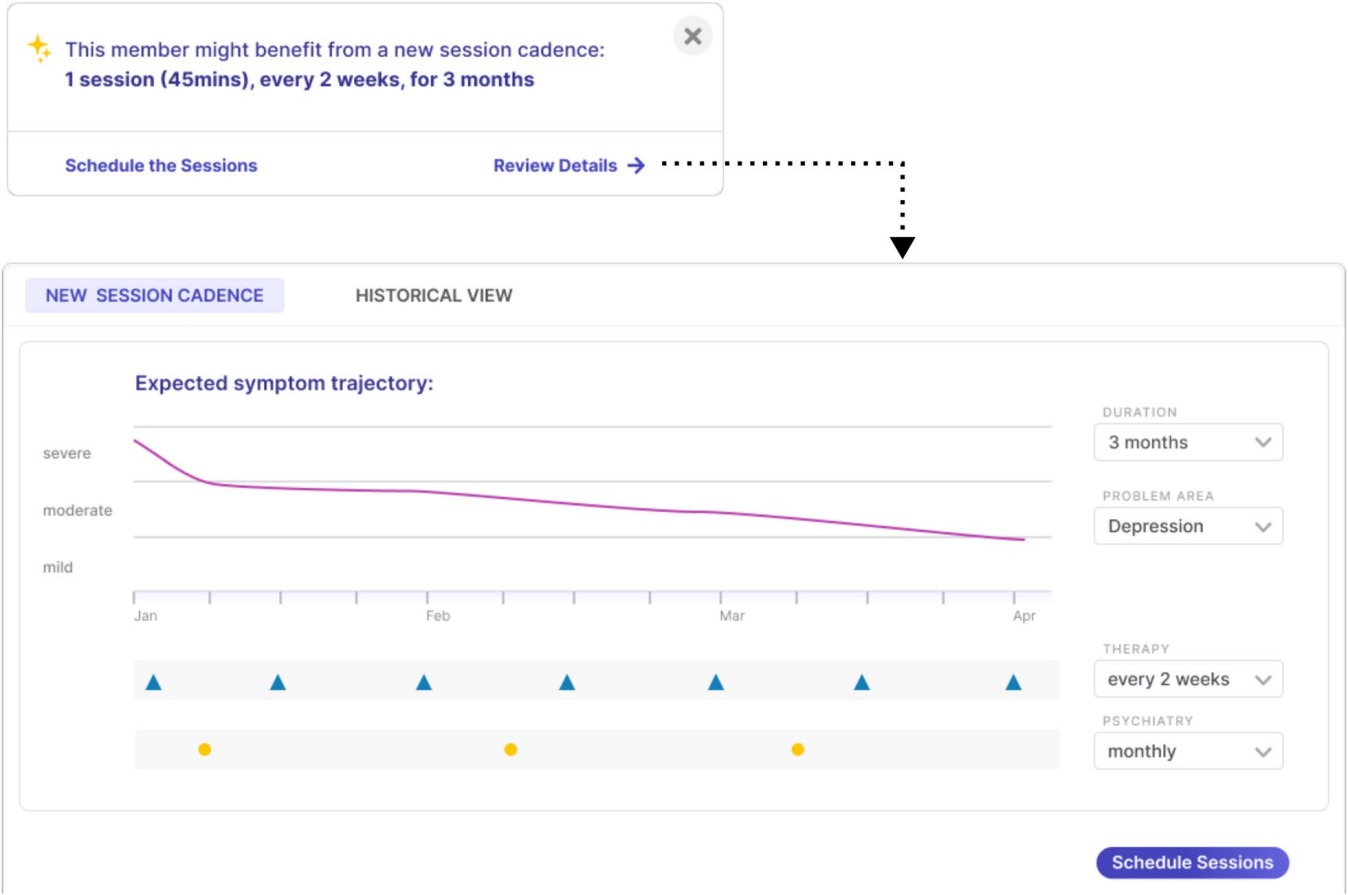
Conceptual mock product wireframes addressing clinician feedback from our pilot study, demonstrating how to improve usability and integration into a clinical workflow for treatment planning

Figure 5 shows wireframes of the initial dashboard used to enable clinicians to interface with the simulation portion of the presented modeling framework, enabling them to test hypothetical treatment schedules for their patients. Further, Fig. 6 highlights mock product concepts resulting from the clinician feedback demonstrating current thinking of what type of tool will be taken into future evidence generation studies.

The total number of patients that had documented chart reviews was 126, with an average (interquartile range) of 7.1 (3–10) per clinician. Table 5 provides results for the clinician use part of the survey. Most providers (n = 14; 63%) responded “A few times” when asked how often they viewed the dashboard per patient. Six clinicians (n = 6, 27%) viewed the dashboard before or after each session with their patient, and two clinicians (n = 2, 9%) viewed the dashboard only once per patient. Providers reported that reviewing the dashboard prior to each session was time-consuming and instead expressed preference for reviewing the dashboard every couple of sessions, or adding intelligence to the dashboard to tell them when to check in. When it came to interpreting the dashboard, there was a mix of responses. A total of seven clinicians found it difficult to interpret. Seven clinicians (n = 7, 31.5%) reported a neutral feeling about the interpretation difficulty. On the other hand, 8 clinicians reported it was easy or very easy (n = 8; 36%). Upon review of the open-ended responses, six clinicians (n = 6, 27%) expressed that the dashboard was easier to interpret once they had more experience and became more comfortable using it. Some clinicians did not notice a significant change in the estimated symptom trajectory line (e.g. “saturated responses”, see Fig. 3), which made it hard for them to interpret.

**Table 5:** Clinician use of the dashboard

	Frequency	Percentage
Number of times dashboard was reviewed per patient		
Never	0	0%
Only once	2	9%
A few times	14	63%
Before or after each session	6	27%
Level of difficulty to interpret the dashboard		
Very difficult	1	4.5%
Difficult	6	27%
Neutral	7	31.5%
Easy	7	31.5%
Very easy	1	4.5%

Table 6 provides results for the application in clinical practice section of the survey. When asked how often they changed the frequency of sessions during the pilot, five clinicians (n = 5, 22.5%) said “never” and 10 (n = 10, 45%) said “rarely.” These clinicians reported that they often would rely more heavily on their clinical judgment rather than the dashboard data. With respect to other factors beyond the dashboard that influenced their decision making (data not shown) were the presence of concomitant symptoms (n = 21, 94.5%), presence of an acute stressor (n = 15, 67.5%), and social determinants of health issues (n = 14, 63%). Additionally, some clinicians noted the presence of environmental factors, changes to schedule availability, and patient choice as reasons impacting the decision to alter treatment schedules. When changes were made to the frequency of sessions, 15 clinicians (N = 15, 67.5%) reported that patients were receptive to the change, while one clinician (N = 1, 4.5%) said that one patient was not receptive to change, in which case no changes were made.

**Table 6:** Application of the dashboard in clinical practice

	Frequency	Percentage
How often clinicians made changes in frequency of sessions		
Never	5	22.5%
Rarely	10	45%
Sometimes	6	27%
Often	1	4.5%
Always	0	0%

Importantly, the dashboard facilitated conversations with patients for 16 providers (n = 16, 72.7%). Specifically, six providers (n = 6, 27%) used the dashboard to discuss with patients the option of adding additional services or implementing additional resources. Five providers (n = 5, 22.5%) shared that the dashboard helped facilitate conversations around treatment effectiveness and a discussion on whether utilizing a different treatment approach may be warranted. Other providers stated that it helped provide recommendations to patients on how often to schedule sessions (n = 4, 18%). Finally, for two clinicians (n = 2, 9%) the dashboard facilitated conversations around session adherence for patients who were not consistent in attending treatment.

Six clinicians (n = 6, 27%) found that the dashboard was not helpful. Some of the reasons reported for this included an inability to notice a significant change in the estimated symptom levels and challenges with interpreting the dashboard. In addition, there were some logistical issues that led clinicians to find the dashboard unhelpful including: (i) errors with accessing the dashboard (n = 2, 9%); (ii) an inability to implement changes in session frequencies due to lack of availability with their schedule (n = 2, 9%); (iii) challenges with reviewing the dashboard prior to each session with a patient (n = 2, 9%); (iv) and the dashboard not being user-friendly (n = 2, 9%).

## Discussion

Individual responses to mental healthcare interventions are known to be variable [45–47] and subsequently fuel variable outcomes at the population level; evidence suggests that treatment can be improved when it includes a multimodal approach including measurement-based care (MBC) and psychotherapy [48–51]. However, gaining access to trained mental healthcare professionals is a major barrier to realizing this potential, with average wait times of 6 weeks to three months being reported [52, 53], and over half of the counties in the US being without a single licensed psychiatrist [54]. While increasing the number of trained and licensed mental healthcare professionals is a major need, it is also recognized that adding objective measures of success and optimizing current resources and treatment planning for existing professionals are among the next big challenges and opportunities for mental healthcare [7, 55].

The objective of this work was to leverage observational clinical data from a learning healthcare system employing measurement-based care (MBC) to develop an objective computational framework capable of identifying temporal links between treatment schedules and fluctuations in depressive symptom severity measures (symptom dynamics), which could subsequently be used to optimize treatment planning in certain cases. We also sought to deploy the initial framework in the same healthcare system at a very small scale, as an exploratory clinical quality improvement (QI) pilot, to gain experience deploying rough prototypes into a real-world clinic and generate learnings in support of evidence generation study design and product development.

Conceptualizing mental healthcare treatment sessions as a type of therapeutic input (‘dose’) presumed to have a measurable impact on symptom levels is a novel approach to modeling in healthcare. We adapted principles from systems dynamics modeling focused on linking the time-course of physiological responses to medication to the time-course of that medication in the body. While not a perfect analogue, the proposed framework utilizes a hierarchical general systems dynamics model and was able to resolve temporal symptom dynamics from historical treatment sessions of various types. To our knowledge, this is the first application of systems dynamics models to identify the time-course and impact of individual mental healthcare services on a specific symptom at an individual (and population) level.

The observation that depressive symptom severity dynamics were often capable of being resolved through session schedules alone is noteworthy; however, a wide range of model performance—and applicability of model assumptions at the individual level—was observed. While there were some individuals whose symptom dynamics were well-resolved by the model, other profiles were not captured well: This was not surprising by itself as there are undoubtedly external factors, life events, etc. driving why an individual’s depressive symptom severity may fluctuate.

We posit that in the cases where symptom dynamics do not track with session schedules (poor model fits), knowing this as early as possible presents opportunities to assess clinical plans, and when symptom dynamics are well characterized by treatment schedules, this should be interpreted as a potentially transient relationship and used to design alerts and planning for the near- to mid-term and should always be re-evaluated across model iterations. However, both of these scenarios offer earlier and more proactive, data-driven, opportunities to engage patients regarding their care, goals, and progress.

It is not proposed that the current framework serve as a mechanistic description of symptom trajectories; rather it serves as an objective tool, leveraging both current and historical data, to inform clinical decision making. In fact, one of the primary benefits of this approach is the ability to incorporate new data as it is generated to re-evaluate and refine treatment planning. Inevitably, as more data is collected, the relationship between symptom dynamics and treatment schedules will shift. As discussed in the Methods section, this nuance is a primary driver for why traditional model evaluation is largely not appropriate for this application and why fit-for-purpose model assessment at the individual level leveraging the IWRES measure was adopted. That being said, in the context of a learning healthcare system, this nuance does not provide a challenge to impact, instead it provides the ability to adjust individual planning in near real-time, rather than after completion of full treatment courses. Although such healthcare systems are not commonplace as of yet [56], early clinical research such as the modeling framework presented here, benefits greatly from the ability to deploy early QI initiatives in these ecosystems, enabling rapid prototyping and product development that is truly born in the clinic. Further, as there is a current lack of consensus on the best way to deploy technologies such as this in real-world clinics, with ranging concepts like the ‘digital clinic’ [57–59] and the ‘trier treatment navigator’ [60, 61] being presented in the research literature, reports from real-world QI initiatives such as this are valuable.

It is important to acknowledge that introducing a new tool, which is both innovative and first of its kind, can elicit skepticism and concern to providers, who all strive to provide the highest quality care. For the presented test pilot, we emphasized that the current modeling framework is not meant to replace a provider’s clinical judgment, but rather serve as an objective data point that can be used to inform treatment—it is up to the provider to consider all factors that the dashboard is not taking into account, including acute stressors, social determinants of health issues, the therapeutic relationship, the stage and goals of treatment, the therapeutic approach, and more. An important topic that arose during the QI pilot was a concern that looking back to see patients who showed slower (or lack of) progress could be interpreted as a reflection of a provider’s quality. It cannot be stated strongly enough that the presented framework is not a clinical assessment tool. And while there will inevitably be new ideas and insights when looking retrospectively at data through a new lens, there must be concurrent commitments to psychological safety amongst clinical teams and a true embrace of learning and progress at all levels for these initiatives to be successful. As a novel learning healthcare system, we are able to deploy this (and other) innovations with sanctity and commonality of intent. By the end of the pilot, providers who used the dashboard more often were comfortable interpreting ‘saturated responses’ as an opportunity for discussion with their patients to understand any obstacles in treatment and evaluate whether to utilize a different therapeutic approach, which we felt was one of the most positive findings of the pilot.

The QI pilot demonstrated a range of experiences and comfort levels using a novel decision support tool for treatment planning. It was known going into the pilot that there would be much feedback from clinicians on improving the user experience and streamlining the technology into the existing clinical record platform, which was received and utilized by the design team to plan development criteria and develop formal mockups (see Fig. 6).

Some of the clinicians expressed difficulty interpreting the dashboard, while those who found it easiest to interpret were the ones who were able to interact more with it and were able to take advantage of one-on-one meetings with the research team. We interpret this as a positive signal that even at this early stage, more exposure appears to be leading to more comfort. And while the user facing portion of the modeling framework indeed lacks distillation of the content into clinically digestible or actionable pieces, one of the goals of the pilot was to identify how the technology may be used in a naturalistic setting in order to facilitate and direct these development efforts.

In addition to the intended use of informing treatment schedules, the modeling framework became useful for providers in identifying whether there was evidence to support benefit for additional services (coaching, therapy, or psychiatry) to their patient’s care. Clinicians reported that in previous settings such a decision was primarily based on clinical intuition; however, with the support of the data generated by the presented framework, providers were given more data to support decision making, leading to meaningful conversations with their patients. Not only did the dashboard lead to better conversations with patients regarding referrals, it also facilitated conversations about treatment effectiveness, barriers to session adherence, as well as adjustments to goals and clinical interventions.

While the presented framework provides an advancement for utilizing patient-reported outcomes from MBC to optimize treatment schedules and planning, there are limitations to note. First, as discussed previously, the model is not truly mechanistic in the sense that the values driving impact on the symptom dynamics are not physical measurements, but a “latent therapeutic mass”: It is not only possible that individual and population parameter estimates will shift over time, it is likely. One anecdotal example of this would be for patients who enter with severe depressive symptoms and gain coping skills over time and sustain low symptom levels with fewer and fewer sessions (i.e. “responders”): In this scenario, over time the individual fit of the data would shift to reflect longer durations of impact for single sessions. The fact that the individual parameter estimates, and the data generating process itself, are expected to vary across successive model runs as new data emerges does not invalidate model fits or reduce the utility of the framework, rather it is simply a reality that is addressable through regular updating and monitoring of individual fits. To further address this limitation, we are creating methods of automating objective identification of poor model fits at the individual level, which can be used to generate separate triaging strategies: We believe that a critical area of future development will be in optimizing these model assessments for specific outcomes over time. Another potential limitation of the framework is the inability to assess the accuracy of predicted ‘rebounds’ in terms of timing, e.g. if the model predicts the return of severe symptoms following a lapse of 3 months of treatment, how accurate is that? It is unlikely that this area of exploration could ever be validated in real-world clinics as this would create a direct conflict with provisioning high-quality mental healthcare.

Further, it is acknowledged that the current model makes several simplifying assumptions about the complexity of mental health symptom dynamics and the possible impact of services on those symptoms. This is driven by a few primary considerations: First, healthcare delivery in real-world clinics cannot be tailored to support modeling initiatives. Any real-world observational data will present challenges to how much structural model complexity can be supported: For instance, contrast this with a specific example from systems dynamics modeling in the pharmaceutical space where specific data collection strategies need to be designed to capture the expected level of complexity in a process such as absorption of medication from the gastrointestinal tract (e.g., see [62]). This type of experimentation is largely not possible in a real-world healthcare setting, so we must only add model complexity as data naturally allows, which will continue to be an active area of investigation for us. Second, given that this is the first application of a modeling framework to this type of problem, there is no other work or evidence that we are aware of to support additional complexities at the current state; however, as implied above, we believe through application of this framework over time, new opportunities for enhancing the representativeness of these modeling frameworks will present themselves. That being said, we believe developing innovative models and applications with real-world observational data (with appropriate interpretive guidelines and use cases) is a positive innovation that brings objectivity and data into the decision-making process. Such innovations with real-world data are also supported through the increasing acknowledgement in the literature that evidence generated in “gold-standard” randomized controlled clinical trials is often not representative of real world populations [63, 64], as well as concurrent increases in the number of other organizations and regulatory authorities embracing innovations with real-world data [65–67].

Finally, the isolation of depression symptom severity as the focus of this framework does not consider co-occurring symptom trajectories that may ultimately impact mental health outcomes. Expansion into other symptoms beyond depression—or the creation of a unified multivariate symptom model—as well as formal feasibility and evidence studies to assess which clinical factors and decision-making domains are impacted by this technology, are needed and are actively being pursued. However, it is important to consider that this technology is developed to be a window by which clinicians can gain additional perspective to the treatment history for their patients and adapt decision making accordingly. So, while this model may be naïve to other symptoms or lifestyle considerations impacting healthcare outcomes, as with any decision support tool its output should not be consumed in isolation—rather, it is intended to be consumed by clinicians and scientists working together to advance the application of technology in mental healthcare. And although the specific model estimations on the current data set are not expected to generalize directly across healthcare systems leveraging different symptom severity reports (e.g., PHQ-9 or HAMD for depression), modeling symptom severity on a relative min–max scale (as was done in this work) opens the door to the possibility of utilizing these results (or others) as informative priors for subsequent modeling efforts, making the application of this framework across distinct healthcare systems another exciting possible area of future application.

## Conclusions

This work presented results from a retrospective analysis of real-world observational clinical data that resulted in a computational framework for personalizing mental healthcare service utilization. In many cases, longitudinal patterns of patient-reported depression symptom severity could be temporally linked to historical treatment data in the form of mental healthcare service utilization. The strength of evidence supporting sensitivities to treatment schedule changes on depressive symptoms varied at the individual level, which prompted development of objective evaluation criteria for triaging model fits for utility.

The framework was subsequently deployed in a learning healthcare system at a small scale to gauge early signals of strengths and limitations for utility in clinical decision making. The ability to report feedback from utilization of developing digital technologies in real-world mental health clinics is valuable to the literature given the evolving landscape and experiences of deploying sophisticated decision support tools. In the pilot, impressions for utility and general comfort for the technology were mixed, which was expected given the literature and findings to date. However, in addition to helping guide decision making, the presentation of historical data and ability to investigate sensitivities to treatment schedules enabled multiple opportunities for clinicians to positively interact with their patients. Better user-interface and automated triaging were requested to lower the barrier of entry to the technology in future studies.

Despite perceived limitations with observational data in many research communities, real-world impact is often gauged in observational data and this work demonstrates a conceptual advancement in leveraging measurement-based care (MBC) to personalize treatment planning at the individual level in near real-time, as well as early pragmatic clinical signals for scaling up and enhancing such solutions.

While formal efficacy studies are being planned currently, work such as this may provide inspiration and learnings for development of new algorithms and objective computational frameworks for optimizing clinical care planning and resource support as more institutions adopt MBC frameworks.

## Supplementary Information

**Additional file 1.** Technical model and estimation details.

**Additional file 2.** NONMEM model code file for estimation.

**Additional file 3.** Model diagnostics and support level determination.

**Additional file 4.** Clinical pilot feedback form.

## Data Availability

Source code and summary variables for the data have been provided. Public provisioning of Mindstrong healthcare data is not permissible per privacy policy and terms of service; however, individual requests for data may be made to the corresponding author and will be triaged to an appropriate privacy officer for consideration on a case-by-case basis. The modeling framework presented here has been filed with United States Patent Office as Provisional Application No. 63/400,770, but may also be.
