# Supplementary material for: A framework for precision “dosing” of mental healthcare services: algorithm development and clinical pilot
Source: Int J Ment Health Syst. 2023 Jul 5;17:21. doi: 10.1186/s13033-023-00581-y (PMC10320892; doi:10.1186/s13033-023-00581-y)
Supplement: Supplementary file 1 — Additional file 1. Technical model and estimation details. [file 13033_2023_581_MOESM1_ESM.pdf]

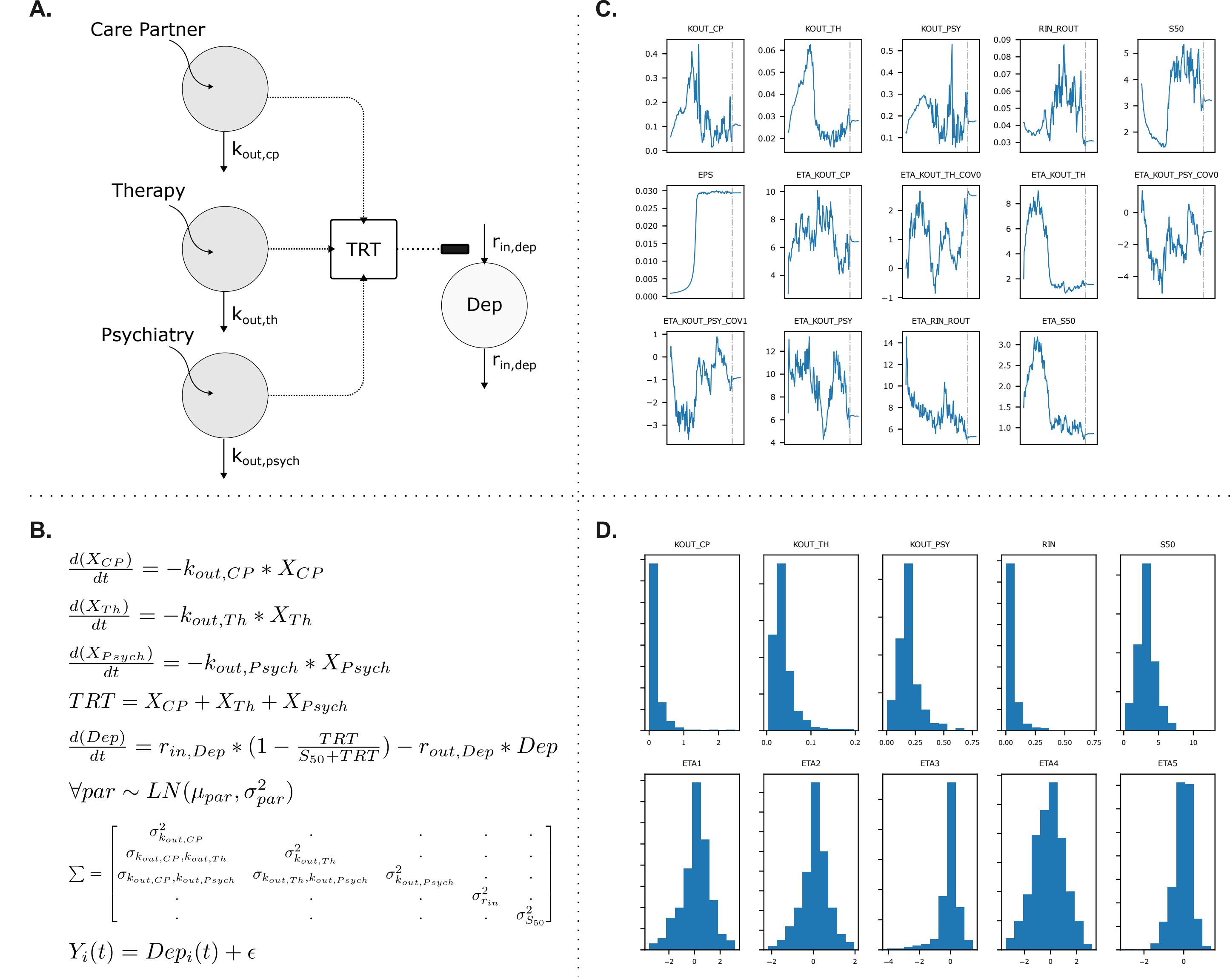

Figure XX: Modeling details for one version of leveraging indirect response models to capture population dynamics of responses to different types of mental healthcare services within a virttual learning healthcare ecosystem. **A**: Symptom dynamics (Dep compartment) are modeled as having zero-order production and first-order elimination ($r_{in,dep}$ = $r_{out,dep}$), where the values of this compartment are patient-reported symptom severity scores. The assumption being that symptoms are generated and eliminated at some quasi-equilibrium when entering into the model. For this work, only members who enter treatment with severe depression are captured. The simplifying assumption that generation and elimination rate constants are equal allows a more parsimonious model and has the implication of equilibrium at the initial starting point. All of these assumptions can be challenged when modeling future data.The structural model is set up to take session events as unit inputs into a latent service compartment. The choice of using a unit input as 'mass' into the latent space is arbitrary and can be challenged with the data over the course of modeling to gauge improvements in the fit of the data. Each service compartment has a first-order elimination rate constant ($k_{out,*}$), which is a convenient starting point for modeling as it ensures values greater than zero. Each service compartment is combined into a combined latent treatment 'mass' (TRT), which is used to drive inhibition of depressive symptoms. **B**: Equations for the structural and statistical error model. For the indirect effect of latent treatment mass on depressive symptom production, a simplifying assumption is made such that the maximum theoretical effect of treatment is 1 (complete inhibition) such that the familiar E_{max} term for the hill equation is missing (fixed to 1). The sensitivity parameter (S_{50}) remains and represents the point at which symptoms begin to rise following the TRT 'mass' falling below that value. All parameters in the model are assumed to follow log normal distributions at the population level, and we have included covariance terms for the service compartment eliminations so that population information can inform likely values for members with unobserved session types. Finally, the population residual error model is assumed to be additive as an initial starting point. **C**: Trace plot for the algorithm estimation process, the stochastic approximation expectation-maximization algorithm was leveraged. the vertical line represents the end of teh 'burn in' phase and the start of the EM phase. From the trace plot it is apparent that the algorithm was able to search the parameter space and does not appear to have been stuck in local minima. **D**: histograms of population values for the estimated parameters. The top row of histograms represent the estimated value of the parameter across the population while the bottom row represents the fit of the random variable. In the presented model, the random variables should be normally distributed with mean zero and estimated variance: The population histograms appear reasonably normally distribution, although kout,psy appears left skewed and the sensitivity S50 parameter may have two data generating processes given the wide mass at zero. These observations represent opportunities to further develop the model over time. However, it should be noted that the intended use of this model is to identify relative levels of effective treatment based on historical data, and it is the individual fits that drive subsequent action, and minor discrepancies in population distributions do not generate significant concern regarding the individual fits. it should also be noted that given the latent nature of the modeling space and the observational nature of the data, individual fits are expected to shift over time and parameter values for one individual may change as new data arises - one example of this would be a patient moving from a 'treatment resitant' period, to a 'treatment responsive' period.
