# Supplementary material for: A framework for precision “dosing” of mental healthcare services: algorithm development and clinical pilot
Source: Int J Ment Health Syst. 2023 Jul 5;17:21. doi: 10.1186/s13033-023-00581-y (PMC10320892; doi:10.1186/s13033-023-00581-y)
Supplement: Supplementary file 3 — Additional file 3. Model diagnostics and support level determination. [file 13033_2023_581_MOESM3_ESM.pdf]

Model Diagnostics and Confidence Plot

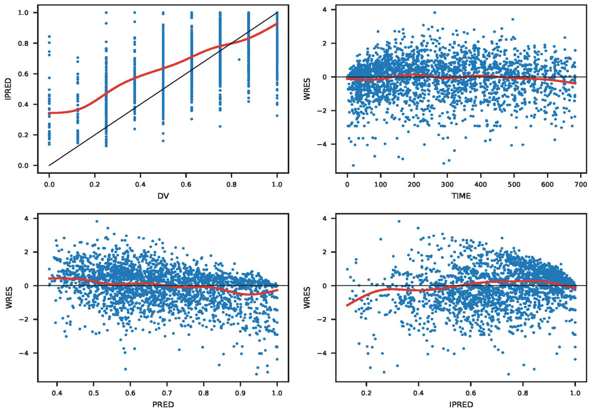

Model visual predictive checks:
IPRED vs DV demonstrates that the model has a systematic bias to overpredict values, however this does not account for the additive residual error which is estimated to have standard deviation around IPRED values of 0,17.

Weighted residuals (WRES) vs time shows that the model is not systematically biased with respect to time. WRES is a normalized version of the residuals, weighted by the defined statistical residual error model.

WRES vs PRED shows similar information as to the IPRED vs DV plot in that the model, after correction for the residual variability still has a slight bias for over-predicting low values and under-predicting high values. It should be noted that this data set has a higher occurrence of values at the extremes, e.g. 0 and 1, than is normally observed in physiological data sets. Data transformations and more logic in identifying appropriate data sets for modeling may be necessary in the future.

WRES vs IPRED shows a classic 'cone' shape indicative that a proportional residual model may be more appropriate. Normally these proportional error models are reversed in the sense that higher observed values tend to have higher levels of variability; however, given the manner in which this population has been selected for the model, there is a systematic bias for higher values, and there may be additional feedback mechanisms necessary for the model to account for additional impact. At the time of writing, the model did not converge with standard proportional error models.

IPRED = individual prediction

WRES = weighted residual

PRED = population prediction

DV = observed data (dependent variable)

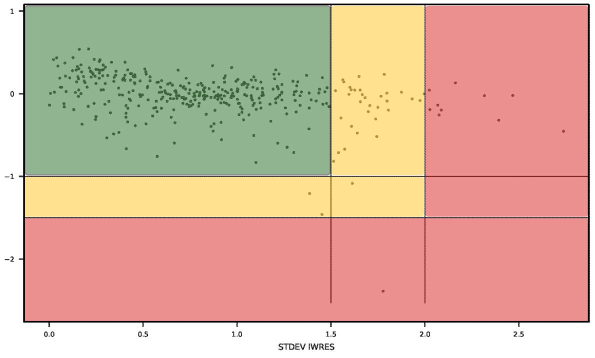

$$IWRES = \frac{DV - PRED}{\sigma}$$

Model Confidence Determinations for Individuals:
With the proposed simplistic additive error model, individual weighted residuals are essentially a measure of a type of z-score around the IPRED value discussed above. Therefore, an IWRES value of 1.0 indicates one standard deviation of the residual error. This plot requires individuals to have at least two observations after the T#IME=0 observation. The average and standard deviation of IWRES values are summarized and used to identify initial cutoffs for confidence in utilizing model fits for treatment planning. Although quantitative scientists could review an individual model fit and determine whether or not the model is a good fit for the data, standardizing decision making is necessary for operationalizing such decision support tools.
