# Supplementary material for: A framework for precision “dosing” of mental healthcare services: algorithm development and clinical pilot
Source: Int J Ment Health Syst. 2023 Jul 5;17:21. doi: 10.1186/s13033-023-00581-y (PMC10320892; doi:10.1186/s13033-023-00581-y)
Supplement: Supplementary file 4 — Additional file 4. Clinical pilot feedback form. [file 13033_2023_581_MOESM4_ESM.pdf]

Session Frequency Pilot Survey

Thank you for your ongoing participation in the Session Frequency Pilot. We kindly ask you to take a moment to complete the following survey to share with us your experience using the Session Frequency Dashboard.

* Required

1. Email *

2. What is your role at Mindstrong? *

Mark only one oval.

- ( ) Therapist
- ( ) Care Partner
- ( ) Psychiatric Prescriber

3. Did you participate in the full pilot? In other words, did you participate for the full duration? *

Mark only one oval.

- ( ) Yes *Skip to question 5*
- ( ) No *Skip to question 4*

Session Frequency Pilot Survey

4. If you responded no to the previous question, please share why you did not participate in the full pilot? *

Mark only one oval.

- ( ) I transferred my members to another provider *Skip to question 5*
- ( ) Other: ______________________

Session Frequency Pilot Survey

5. On a scale from 1-5, how enjoyable was it to work on this pilot? *

Mark only one oval.

- ( ) 1 - Not at all enjoyable
- ( ) 2 - Slightly enjoyable
- ( ) 3 - Moderately enjoyable
- ( ) 4 - Very enjoyable
- ( ) 5 - Extremely enjoyable

6. Would you be interested in participating in a similar pilot again in the future? *

Mark only one oval.

- ( ) Yes
- ( ) No

Session Frequency Pilot Survey

7. Did you review the dashboard throughout the pilot? *

Mark only one oval.

- Yes *Skip to question 8*
- No *Skip to question 9*

Session Frequency Pilot Survey

8. Since starting this pilot (October 2022), how many times have you used the dashboard? *

Mark only one oval.

- Never
- 1-5 times
- 6-10 times
- 11-15 times
- 16-20 times
- 21-25 times
- 25-30 times
- 30+ times

Skip to question 10

Session Frequency Pilot Survey

9. Did you experience any barriers when it came to reviewing the dashboard? *

Mark only one oval.

- I had no barriers
- I could not access dashboard
- I could not interpret the dashboard
- None of my members were eligible
- I did not have time to review the dashboard
- Other: ______________________

Skip to question 10

Session Frequency Pilot Survey

10. How many members did you apply the dashboard with? (here is the link the Member list) *

Type in a the number in the space below

____

11. On a scale from 1-5, how easy was it to apply the dashboard with members? *

Mark only one oval.

- 1 - Very difficult
- 2 - Difficult
- 3 - Neutral
- 4 - Easy
- 5 - Very Easy

12. What method did you use to remind yourself which members were eligible for the pilot? *

Mark only one oval.

- I created a task for myself
- I had it on a note
- I updated the appointment type to my calendar
- I changed the members "nickname" in Care to "pilot"
- Other: ______________________

13. In the dashboard, you were asked to add different frequencies, such as weekly, bi-monthly, monthly, etc. to see the estimated symptom trajectory. On a scale from 1-5, how easy was it to determine the frequencies? *

Mark only one oval.

- 1 - Very difficult
- 2 - Difficult
- 3 - Neutral
- 4 - Easy
- 5 - Very Easy

14. On a scale from 1-5, how easy was it to interpret the dashboard? *

Mark only one oval.

- 1 - Very difficult
- 2 - Difficult
- 3 - Neutral
- 4 - Easy
- 5 - Very Easy

15. Based on your response to the question above, what made it easy or difficult to interpret the dashboard? *

16. On average, how helpful was the dashboard in determining changes to session frequency and treatment planning? *

Mark only one oval.

- 1 - Not at all helpful
- 2 - Unhelpful
- 3 - Slightly unhelpful
- 4 - Neutral
- 5 - Helpful
- 6 - Moderately helpful
- 7 - Extremely helpful

17. Based on your answer to question above, please describe what made the dashboard helpful or not helpful. *

18. On average, how often did you review the dashboard for each of your members? *

Mark only one oval.

- 1 - Never
- 2 - Only once
- 3 - A few times
- 4 - Before or after each session

19. When was it most helpful to review the dashboard? *

Mark only one oval.

- Prior to each session with a member
- After each session with a member
- In between sessions
- During session with the member
- Other: ______________________

20. Overall, how often did you make changes to the frequency of sessions with your members after each time you reviewed the dashboard? *

Mark only one oval.

- 1 - Never
- 2 - Rarely
- 3 - Sometimes
- 4 - Often
- 5 - Always

21. Based on your response to the question above, describe what led you to make or not make changes to frequency of session. *

22. What other factors did you consider when determining the change in session frequency? [Check all that apply] *

Check all that apply.

- [ ] Presence of other symptoms aside from depression and/or anxiety
- [ ] Presence of social determinants of health
- [ ] Presence of an acute stressor
- [ ] Other: ____________________

23. Was the dashboard useful in delivering care for other reasons aside from adjusting session frequency? *

Mark only one oval.

- ( ) Yes *Skip to question 24*
- ( ) No *Skip to question 25*

Session Frequency Pilot Survey

24. In what way was the dashboard helpful? [Check all that apply] *

Check all that apply.

- [ ] It was helpful to review past session frequencies
- [ ] It was helpful to determine if the member would benefit from added services (referring to therapy, care partners, and/or psychiatry]
- [ ] It was helpful to open a conversation with the member around their overall treatment and progress
- [ ] Other: ______________________

Session Frequency Pilot Survey

25. How many times did you add an additional service based on the dashboard data? *

Insert a number

____

Session Frequency Pilot Survey

26. Did the dashboard inform any of your conversations with your members? In other words, after reviewing the dashboard, did it spark any conversations with your members? *

Mark only one oval.

- ( ) Yes *Skip to question 27*
- ( ) No *Skip to question 28*

Session Frequency Pilot Survey

27. In what way did it inform your conversations? *

____
____
____
____
____

Skip to question 29

Session Frequency Pilot Survey

28. What, if anything, deterred you from using the dashboard to inform your conversations with your members? *

____
____
____
____
____

Session Frequency Pilot Survey

29. How did your members respond to any changes made to their session frequency? *

Mark only one oval.

- I didn't change the session frequency for any of my members
- Members were receptive to the change
- Members were NOT receptive to the change
- Members were confused
- Other: _______________

30. If you made any changes to member's care based on what you saw on the dashboard aside from the session frequency (i.e. you added a new service like therapy, care partner, psychiatry, or you discussed changing the treatment approach), how did your member respond? *

Mark only one oval.

- I didn't make any changes
- Members were receptive to the change
- Members were NOT receptive to the change
- Members were confused
- Other: ______________________

31. How did you talk to your member about any changes made? *

____

____

____

____

____

32. Did you notice any improvements in members' symptoms after making any changes to the session frequency? *

Mark only one oval.

- Yes *Skip to question 33*
- No *Skip to question 34*

Skip to question 24

Session Frequency Pilot Survey

33. Describe what improvements you noticed in your members. *

Skip to question 34

Session Frequency Pilot Survey

34. What do you need to make this dashboard more helpful in informing treatment planning? *

35. What about this dashboard was most helpful? *

36. What about this dashboard was least helpful? *

37. Do you have any recommendations on how to make it better? *

38. Any other feedback you want to share with us? *

This content is neither created nor endorsed by Google.

Google Forms
